# Biofilm Formation by Hospital-Acquired Resistant Bacteria Isolated from Respiratory Samples

**DOI:** 10.1007/s44197-024-00215-7

**Published:** 2024-04-02

**Authors:** Hila Ben-Amram, Maya Azrad, Jackie Cohen-Assodi, Adi Sharabi-Nov, Shimon Edelstein, Keren Agay-Shay, Avi Peretz

**Affiliations:** 1 Azrieli Faculty of Medicine, Bar-Ilan University, Safed, Israel; 2 The Clinical Microbiology Laboratory, Ziv Medical Center, Affiliated with Azrieli Faculty of Medicine, Bar Ilan University, Safed, Israel; 3 The Clinical Microbiology Laboratory, Tzafon Medical Center, Affiliated with Azrieli Faculty of Medicine, Bar Ilan University, Safed, Israel; 4 Ziv Medical Center, Safed, Israel; 5 The Infectious Diseases, Ziv Medical Center, Affiliated with Azrieli Faculty of Medicine, Bar Ilan University, 1311502 Safed, Israel

**Keywords:** Hospital-acquired resistant infections, Risk factors, Biofilm, Antibiotic-resistant bacteria

## Abstract

**Background:**

Hospital-acquired resistant infections (HARI) are infections, which develop 48 h or more after admission to a healthcare facility. HARI pose a considerably acute challenge, due to limited treatment options. These infections are associated bacterial biofilms, which act as a physical barrier to diverse external stresses, such as desiccation, antimicrobials and biocides. We assessed the influence of multiple factors on biofilm production by HARI -associated bacteria.

**Methods:**

Bacteria were isolated from samples of patients with respiratory HARI who were hospitalized during 2020–2022 in north Israel. Following antibiotic susceptibility testing by disc diffusion or broth microdilution, biofilm formation capacities of resistant bacteria (methicillin-resistant staphylococcus aureus, extended spectrum beta-lactamase-producing *Escherichia coli* and *Klebsiela pneumonia*, and multidrug-resistant *Pseudomonas aeruginosa* and *Acinetobacter baumannii*) was assessed using the crystalline violet staining method. Data regarding season, time to infection, bacterial species, patient age and gender, year, and medical department were collected from the patient medical records.

**Results:**

Among the 226 study isolates, *K. pneumonia* was the most prevalent (35.4%) bacteria, followed by *P. aeruginosa* (23.5%), and methicillin-resistant staphylococcus aureus (MRSA) (21.7%). A significantly higher rate of HARI was documented in 2022 compared to 2020–2021. The majority of isolates (63.3%) were strong biofilm producers, with *K. pneumonia* (50.3%) being most dominant, followed by *P. aeruginosa* (29.4%). Biofilm production strength was significantly affected by seasonality and hospitalization length, with strong biofilm production in autumn and in cases where hospitalization length exceeded 30 days.

**Conclusion:**

Biofilm production by HARI bacteria is influenced by bacterial species, season and hospitalization length.

## Background

Hospital-acquired resistant infections (HARI) are defined as infections, which develop 48 h, or more after admission to a hospital [1]. HARI pose a considerably acute challenge, due to limited treatment options [2].

The injudicious and more widespread use of antibiotics is a critical factor driving the development of bacterial antibiotic resistance [3] and increase in treatment failures rates [4]. The Centres for Disease Control and Prevention (CDC), Infectious Diseases Society of America, World Economic Forum and World Health Organization (WHO) have all flagged antibiotic resistance as a global public health concern [5]. Gram-negative bacteria are responsible for more than 30% of HARI, and dominate in cases of ventilator-associated pneumonia (VAP) [6].

Hospital-acquired pneumonia (HAP) and VAP are significant health problems worldwide [7]. HAP is currently the main cause of death among nosocomial infections in critically ill patients, with an incidence of 5–10 cases per 1000 hospital admissions. The estimated mortality rate of HAP is 20–30%, and has been reported to be up to 50% in VAP [8]. The most frequently associated Gram-negative bacteria with HAP/VAP are *Pseudomonas aeruginosa* (*P. aeruginosa*), *Acinetobacter baumannii* (*A. baumannii*), and *Enterobacteriales* [9]. Among Gram-positive pathogens causing HAP/VAP, *Staphylococcus aureus* (*S. aureus*) is one of the most common, with an associated mortality rate of approximately 30–40% [10].

Multi-drug resistant (MDR) Gram-negative bacteria are defined as those resistant to at least three different families of antibiotics. At present, β-lactam drugs are commonly prescribed for bacterial infections worldwide and account for almost 65% of antibiotic usage [11]. Intensive use and misuse of β-lactam antibiotics both in human and in veterinary medicine has led to the spread of extended spectrum β-lactamase (ESBL)-producing bacteria [12].

In addition to antibiotic resistance, biofilm production also contributes to bacterial virulence [13], and has been linked to up to 80% of bacterial infections [14]. A biofilm is defined as a polymicrobial aggregate which attaches to other aggregates and/or to surfaces. Biofilms can be found in a wide variety of settings, including on medical devices, where they can act as a physical barrier to diverse external stresses, such as desiccation, antimicrobials and biocides. Additionally, the biofilm enables evasion of the host immune response and nutrient preservation [15].

The current study investigated the bacterial species distribution in respiratory samples of patients diagnosed with HARI between the years 2020–2022 in ICU and internal medicine departments. Additionally, the impact of seasonality, bacterial characteristics, hospitalization length, age, gender, and hospitalization year on biofilm production in HARI pathogens was assessed.

## Methods

### Study Population

Clinical isolates were recovered from respiratory samples (sputum, broncho-alveolar lavage) of adult patients (> 18 years) hospitalized in the ICU and internal medicine departments at Ziv Medical Center (ZMC, Safed) and Tzafon Medical Center (TMC, Poriya) during 2020–2022, as part of the routine medical care at these medical centres. These isolates included methicillin-resistant *Staph aureus* (MRSA), extended beta lactamase producing *Escherichia coli* and *Klebsiela pneumonia* (ESBL *E. coli and ESBL K. pneumonia*) and multi drug resistant *Pseudomonas aeruginosa* and *Acinetobacter baumannii* (MDR *P. aeruginosa* and MDR *A. baumannii*). The study was approved by the ZMC and the TMC Helsinki ethics committees (approval No. 0068-19-ZIV, 0002-20 POR), which waived the need for patient consent.

### Sample Collection and Bacterial Isolation

Bacterial isolates were identified using matrix-assisted laser desorption ionization-time of flight (MALDI-TOF) (Bruker Daltonics, Bremen, Germany) and antimicrobial susceptibility was determined by Vitek 2 (bioMérieux, Inc., Hazelwood, MO, USA).

### Determination of ESBL Production

To identify ESBL production, *E.coli* and* K. pneumonia* strains were grown at 37 °C for 18–24 h before susceptibility tests. Following incubation, several colonies were suspended in saline to a turbidity of 0.5 McFarland. The suspension was seeded on a Muller–Hinton (MH), (BD Diagnostics, Sparks, MD, USA) agar plate, after which, cefotaxime, cefotaxime/clavulanic acid, ceftazidime and ceftazidime/clavulanic acid antibiotic discs (BD Diagnostics) were placed on each agar plate. Plates were then incubated at 35 °C for 16–20 h. An increase in zone diameter of ≥ 5 mm between cefotaxime and cefotaxime/clavulanic acid, or ceftazidime and ceftazidime/clavulanic acid was considered ESBL-positive.

### Further Variables

The following data were collected from the patient medical records: age, gender, hospitalization year, time between hospitalization and HARI diagnosis (48 h–10 days, 11 days–30 days, or ≥ 31 days) and season of hospitalization (Winter: December through March, Spring: April through May, Summer: June to September, and Autumn: October until November).

### Detection of Biofilm Formation

Biofilm formation capacity was assessed using the crystalline violet staining method. Bacteria were inoculated on MH plates (BD Diagnostics) and incubated at 37 °C for 24 h. Then, colonies were suspended in 1 mL sterile brain heart infusion (BHI) (Hy Laboratories, Rehovot, Israel) to a turbidity of 0.5 McFarland’s. Bacterial suspensions were transferred to 96-well plates in triplicates (200 μL per well). Sterile LB broth served as a negative control. The plates were incubated at 37 °C for 48 h. Following incubation, floating bacteria were removed by rinsing the plates 3 times with 200-μL sterile distilled water. Then, 200 μL 99% methanol were added to each well for 15 min; the wells were then rinsed and dried. Crystalline violet dye (1%, 200 μL/well) was added for 20 min, plates were washed 3 times with sterile distilled water, and dried at room temperature. Then, 95% ethanol (200 μL per well) was added for 10 min to fully dissolve the crystalline violet. Absorbance (OD) was read at 595 nm a Multiskan FC microplate reader (Thermo Scientific, Waltham, USA). Biofilm-forming capacity was classified as weak (OD ≤ 2*ODc) or strong (OD > 2*ODc).

### Statistical Analysis

A descriptive statistical analysis was performed to determine the distribution of the research variables across the study population. Categorical variables are presented as count and percent. The statistical significance of the difference between categories was assessed using the Chi-squared test. Generalized linear models with binary family and log link function were used to evaluate the differences in odds ratios (ORs) and 95% CIs of the biofilm-production capacity and the independent variables (season, time to acquisition, bacteria type, age, gender, year and medical department).

All statistical analyses were performed using SPSS software version 25 (IBM) and Office EXCEL 2016 software, with statistical significance threshold of *p* < 0.05.

## Results

### General Characteristics of Study Isolates

**Fig. 1:**
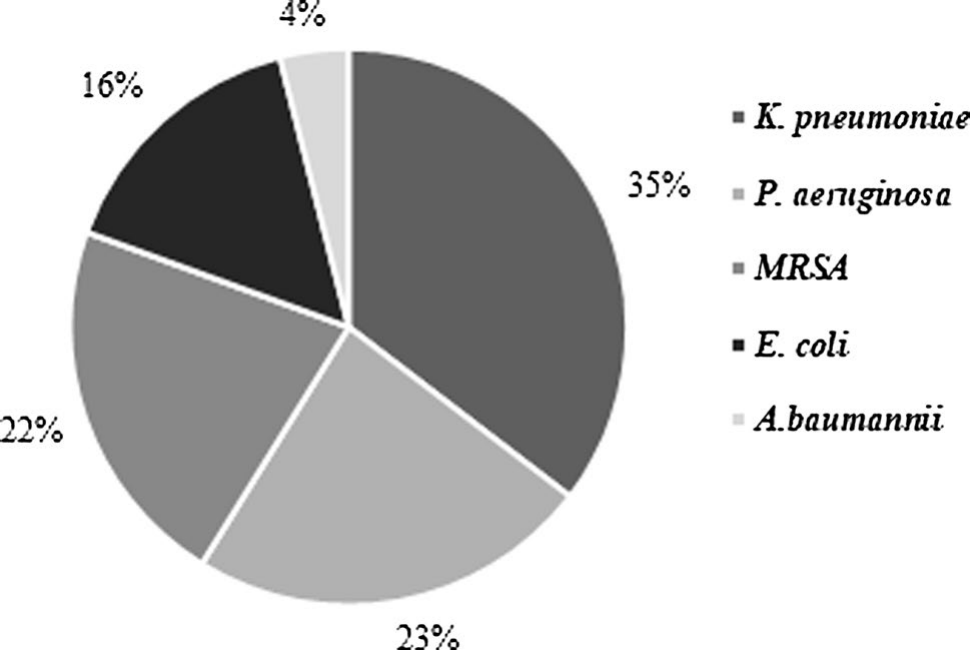
Distribution of species isolated from clinical respiratory samples (n = 226) of patients with hospital-acquired resistant infection in the years 2020–2022

A total of 226 isolates were collected during the study. Of these, the most prevalent bacterial species was ESBL-*K. pneumonia* (80 (35.4%)), followed by *P. aeruginosa* (53 (3.5%)), MRSA (49 (21.7%), ESBL-*E.coli* [35 (15.5%), and *A. baumannii* 9 (4%)] (Fig. 1).

**Fig. 2:**
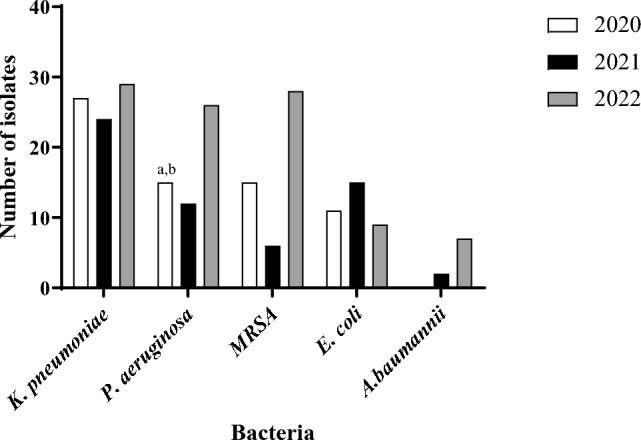
Bacterial distribution among patients with respiratory HARI between the years 2020–2022

A significant increase in the prevalence of HARI was documented in 2022 (n = 99) compared to 2020 (n = 68) and 2021 (n = 59) (*p* < 0.05) (Fig. 2).

*Acinetobacter baumannii* prevalence significantly increased over the study years, from a prevalence of 0% in 2020 to 7.1% in 2022. MRSA prevalence significantly increased between 2021 (12.2%) and 2022 (57.15%), while *E. coli* prevalence decreased between 2021 (42.9%) and 2022 (25.7%) (*p* < 0.05).

**Fig. 3:**
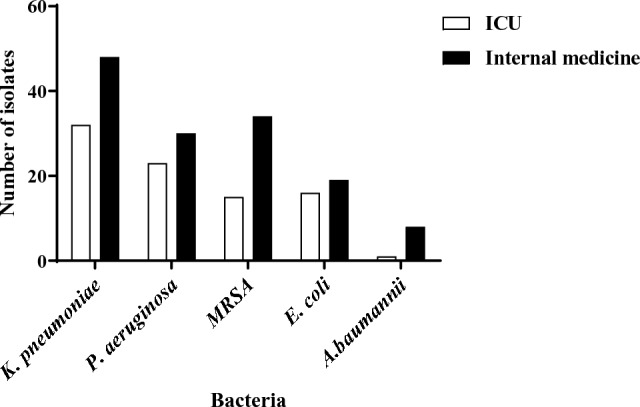
Distribution of HARI-bacteria in ICU and internal medicine wards at ZMC and TMC during the years 2020–2022

The majority of HARI isolates were recovered from patients hospitalized in internal medicine departments (139 (61.5%)) (Fig. 3).

Furthermore, each of the identified bacterial species was more prevalent in internal medicine wards compared to the ICU, however, the differences were not statistically significant.

**Fig. 4:**
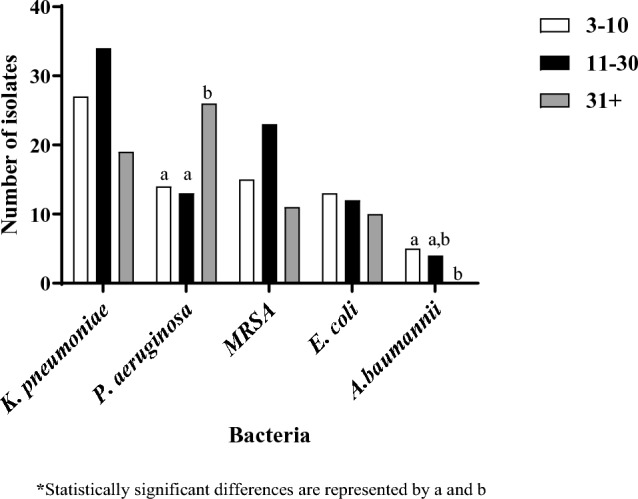
Bacterial species distribution by time to acquisition

Bacterial infections were most frequently acquired between days 11 and 30 (86, 38.1%), followed by 3–10 days (74, 32.7%) or 31 or more days (66, 29.2%) after admission. *A. baumannii* acquisition was significantly more frequent 3–10 days after admission, while *P. aeruginosa* acquisition was significantly more frequent at 31 days or more after admission (Fig. 4).

### Biofilm Production by HARI-Associated Bacteria

**Fig. 5:**
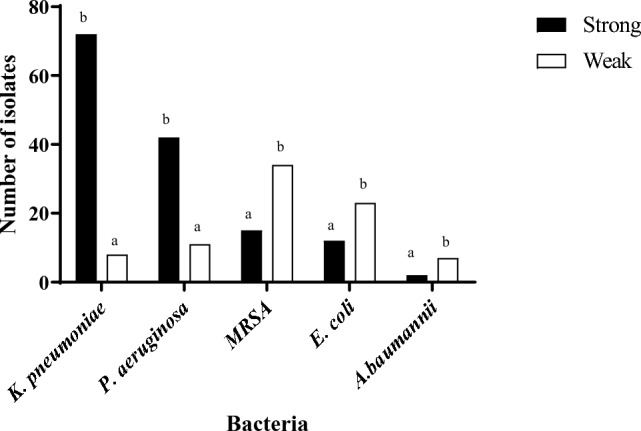
Biofilm production in different HARI-bacteria isolated during the years 2020–2022

Overall, 143 (63.3%) isolates were strong biofilm producers (Fig. 5).

Comparison of biofilm strength across bacterial species found the highest prevalence of strong biofilm producers among *K. pneumonia* (n = 72, 50.3%), followed by *P. aeruginosa* (n = 42, 29.4%), MRSA (n = 15, 10.5%), *E. coli* (n = 12, 8.4%) and *A. baumannii* (n = 2, 1.4%) (*p* < 0.001) isolates. Interspecies comparisons found that 90% of the ESBL-*K. pneumonia* isolates and 79% of the *P. aeruginosa* isolates were strong biofilm producers. In contrast, most MRSA, *E. coli* and *A. baumannii* isolates were weak biofilm producers.

### Factors Influencing Biofilm Formation

Seasonality significantly influenced the ability to produce biofilm (*p* < 0.01). In the autumn season, the prevalence of strong biofilm producer bacteria was significantly higher compared to other seasons (OR 4.15) (Table 1).

**Table 1:** Risk for strong biofilm production by independent patient, bacterial and environmental variables

Variable	Category	OR	95% CI	*p*
Age	Years	0.99	0.97–1.02	0.512
Gender	Male	1.00		
	Female	1.33	0.60–2.97	0.483
Year	2020	1.00		
	2021	0.77	0.29–2.04	0.603
	2022	0.84	0.34–2.09	0.713
Department	ICU	1.00		
	Internal A	1.88	0.87–4.02	0.106
Season	Winter	1.00		
	Spring	1.78	0.64–4.95	0.269
	Summer	1.06	0.38–2.72	0.958
	Autumn	4.15	1.45–11.85	**0.008**
Time to acquisition	3–10 days	1.00		
	11–30 days	2.56	1.08–6.09	**0.034**
	≥ 31	2.98	1.16–7.69	**0.024**
	*Trend*			**0.039**
Species	*A. baumannii*	1.00		
	*E. coli*	3.70	0.49–28.09	0.206
	*K. pneumonia*	81.97	10.42–645.15	< **0.001**
	*P. aeruginosa*	26.07	3.42–198.5	**0.002**
	MRSA	1.98	0.30–13.32	0.481

*ICU* intensive care unit, *MRSA* methicillin-resistant *Staphylococcus aureus*

Bold indicate statistically significant results

**Fig. 6:**
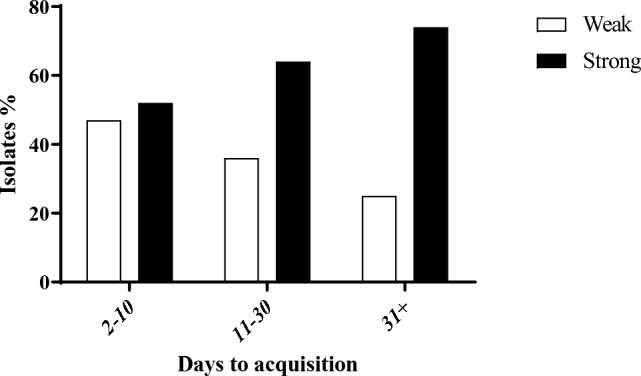
Acquisition of weak and strong biofilm producer bacteria at three hospitalization length groups

Biofilm formation intensity significantly increased with hospitalization length, with the strongest biofilm producers identified in samples from patients hospitalized for 31 or more days (74.2%; OR = 2.98, *p* = 0.024) (Table 1). At 3–10 days, 52.7% of the isolates were strong biofilm producers, while at 11–30 days, 64% of the isolates were strong biofilm producers (*p* < 0.05). In contrast, risk of acquisition of weak biofilm producers decreased with hospitalization length (Fig. 6).

Age, gender, hospitalization year and hospitalization departments showed no correlation with the intensity of biofilm production in the examined bacteria (Table 1).

## Discussion

This study profiled bacterial isolates collected from hospitalized patients with respiratory HARI, and focus on factors affecting biofilm production.* K. pneumonia* was the most prevalent pathogen, followed by *P. aeruginosa*, similar findings were reported by Lev et al., who showed that 57% of the isolates recovered from 112 respiratory samples were *K. pneumonia* [16]. Meta-analysis that screened 57 publications showed an increase in the prevalence of pathogens causing HARI pneumonia between the years 2011–2021. These data align with the current demonstration of an increase in HARI prevalence in 2022 compared to 2020–2021. A recent review comparing the incidence of HARI between the years 2019 and 2020 found some increases in the numbers of acquired infections over time [17]. These increases may be a result of the COVID-19 pandemic. Shabaklo et al. reported on a lower incidence of MDR infections during the first wave of COVID-19, compared to the late pandemic periods. This may be due to the more widespread use of personal protective equipment and stronger adherence to infection control procedures in the earlier stages of the pandemic [18]. In our study, the most significant increase in HARI between the years 2020 to 2022 was of infections caused by *A. baumannii*, which was reportedly the most prevalent bacterial coinfections in patients with COVID-19 [19].

The present results showed that most bacterial infections were acquired between days 11 and 30 of admission. This observation is consistent with reports of pneumonia primarily developing in patients staying in the ward for over 15 days [20] and a 12-fold higher risk of pneumonia in ICU hospitalizations extending beyond 72 h [21].

Overall, 63.3% of the HARI isolates were strong biofilm producers. A recent review of 17 articles characterizing over 2000 Gram-negative isolates, found a high rate (72.4%) of biofilm producers among ESBL-producing strains [22]. In addition, Surgers et al., showed that out of 147 ESBL *K. pneumonia* and *E.coli* (57.1%) were strong biofilm formation compared to 29.5% weak biofilm production and 13.4% who did not produce biofilm [23]. In the present analysis, 90% of the ESBL-*K. pneumonia* isolates were strong biofilm producers. Studies have shown that ESBL-*K. pneumonia* strains have a higher ability to form biofilms compared to non-ESBL-producing strains [24]. As for *K. pneumonia*, 79% of the *P. aeruginosa* isolates were strong biofilm producers. *P. aeruginosa* is considered one of the most common etiological factors of HARI [25] and their biofilms have been significantly associated with MDR [26] and antibiotic resistance [27].

Among the 83 weak biofilm producers in the current study, 41% were MRSA, 27.7% *E.coli*, 13.3% *P. aeruginosa*, 9.6% *K. pneumonia* and 8.4% were *A. baumannii*. The prevalence of weak *E. coli* biofilm producers was in accordance with the outcomes of a meta-analysis of 37 studies conducted between 2000 and 2021 on biofilm production and antibiotic resistance in uropathogenic *E. coli*-positive samples, which found that 38.6% of the *E.coli* isolates were weak producers. A variety of physical factors act onto bacteria in nature. The current work showed a seasonal pattern of biofilm production, with higher biofilm production in autumn. Several previous studies reported seasonality of nosocomial infection [28–31]. A recent study examining the effect of seasonality on HARI incidence found it to increase by 13.1% for every 5 °C rise in temperature [30]. Another study conducted in northern Israel between 2001 and 2008 compared the incidence of *E. coli* in BSI and its association with temperatures in different seasons. *E.coli* BSI was found to be 21% more frequent in summer than in winter, while antibiotic consumption was significantly higher in the winter period [31]. The autumn season in north Israel is characterized by relatively high temperatures (mean = 20 °C). Cam et al. evaluated biofilm production by *Vibrio vulnificus* at different temperatures (24, 30, and 37 °C) and often found 2–3-times more biofilm production at 24 °C as compared to 30 and 37 °C [32]. There is a paucity of information regarding the effect of temperature and seasonality on the ability to form biofilm in samples isolated from clinical sites, in general, and in acquired infections in particular. Further research is still needed in this regard.

A positive correlation was found between biofilm production and hospitalization duration. As hospitalization length increased, the prevalence of antibiotic resistant bacteria also rose. Additionally, antibiotic resistance was associated with biofilm production intensity [33]. Thus, it is possible that the association between hospitalization length and biofilm formation intensity is connected to the antibiotic resistance acquired during hospitalization.

Age, gender, hospitalization year and hospitalization departments had no significant influence on the intensity of biofilm production in the examined bacteria. To the best of our knowledge, the associations between the biofilm production intensity by HARI and these factors have not been reported in Israel or elsewhere.

## Conclusions

This study was performed to assess the influence of various clinical and environmental factors on biofilm production by hospital-acquired resistant bacteria isolated from respiratory samples. *K. pneumonia* and *P. aeruginosa* were identified as strong biofilm producers and seasonality and length of hospitalization were independent risk factors for biofilm production. To prevent biofilm production, patients with these risk factors should be carefully monitored. Further research is needed to evaluate additional risk factors for biofilm production in HARI.

## Data Availability

The datasets used and/or analysed during the current study are available from the corresponding author on reasonable request.
